# Lead and PCBs as Risk Factors for Attention Deficit/Hyperactivity Disorder

**DOI:** 10.1289/ehp.0901852

**Published:** 2010-09-09

**Authors:** Paul A. Eubig, Andréa Aguiar, Susan L. Schantz

**Affiliations:** 1 Department of Comparative Biosciences, College of Veterinary Medicine and; 2 Neuroscience Program, University of Illinois at Urbana-Champaign, Urbana, Illinois, USA

**Keywords:** ADHD, attention, executive function, lead, PCBs

## Abstract

**Objectives:**

Attention deficit/hyperactivity disorder (ADHD) is the most frequently diagnosed neurobehavioral disorder of childhood, yet its etiology is not well understood. In this review we present evidence that environmental chemicals, particularly polychlorinated biphenyls (PCBs) and lead, are associated with deficits in many neurobehavioral functions that are also impaired in ADHD.

**Data sources:**

Human and animal studies of developmental PCB or lead exposures that assessed specific functional domains shown to be impaired in ADHD children were identified via searches of PubMed using “lead” or “PCB exposure” in combination with key words, including “attention,” “working memory,” “response inhibition,” “executive function,” “cognitive function,” “behavior,” and “ADHD.”

**Data synthesis:**

Children and laboratory animals exposed to lead or PCBs show deficits in many aspects of attention and executive function that have been shown to be impaired in children diagnosed with ADHD, including tests of working memory, response inhibition, vigilance, and alertness. Studies conducted to date suggest that lead may reduce both attention and response inhibition, whereas PCBs may impair response inhibition to a greater degree than attention. Low-level lead exposure has been associated with a clinical diagnosis of ADHD in several recent studies. Similar studies of PCBs have not been conducted.

**Conclusions:**

We speculate that exposures to environmental contaminants, including lead and PCBs, may increase the prevalence of ADHD.

With an estimated worldwide prevalence of 5.29% (Polanczyk et al. 2007), attention deficit/hyperactivity disorder (ADHD) is the most common neurobehavioral disorder of childhood. ADHD has an onset at early school age and is characterized by impulsive behavior and inattention, with three main clinical phenotypes: *a*) predominantly inattentive, *b*) predominantly hyperactive-impulsive, and *c*) combined, which includes characteristics of both of the first two phenotypes (American Psychiatric Association 2000).

Aspects of both executive functioning and attention are impaired in ADHD [for a review, see Aguiar et al. (2010)]. Executive function is an interrelated set of cognitive abilities, including working memory, response inhibition, cognitive flexibility, and planning, that are involved in goal-oriented problem solving (Marcovitch and Zelazo 2009). Working memory is the ability to keep information in mind momentarily while using the information to perform an action or while performing an unrelated action (Baddeley 1986). Response inhibition is the ability to either inhibit or interrupt a response, with impaired response inhibition manifesting as impulsivity (Winstanley et al. 2006). Cognitive flexibility (or set shifting) is the ability to reallocate mental resources based on a change in situational demands (e.g., when the rules for successfully completing a task change) (Monsell 2003). Attention consists of several components, including alertness, which is the ability to enter an alert state and immediately focus on important aspects of a situation, and vigilance (or sustained attention), which is the ability to maintain the alert state for a period of time (Nigg and Nikolas 2008). Although all of these neurobehavioral functions are compromised in ADHD children to some extent, the evidence suggests that working memory, response inhibition, and vigilance are affected to a greater extent (Aguiar et al. 2010).

Considerable research has been devoted to identifying factors that contribute to ADHD, but we are still a long way from fully understanding its etiology. ADHD is a highly heritable disorder, with pooled data from twin studies suggesting a heritability of 76% (Smith et al. 2009). Yet emerging evidence indicates that many neurodevelopmental disorders, including ADHD, result from complex interactions of genetic, environmental, and social factors (Pennington et al. 2009). Neuroimaging studies suggest that corticostriatal circuitry in brain regions that have key roles in executive function and attention is altered in ADHD, and several converging lines of evidence point to dysfunctional catecholaminergic signaling as the underlying deficit (Vaidya and Stollstorff 2008). Among the contributing factors, the potential role of toxic exposures, especially those that alter catecholaminergic signaling, deserves special scrutiny because these exposures are, in theory, preventable. Much has been written about ADHD, and a number of recent reviews have discussed the role of environmental exposures (e.g., Banerjee et al. 2007; Nigg 2006; Swanson et al. 2007). However, none has provided an in-depth analysis of the parallels between the performance of contaminant-exposed and ADHD children on specific cognitive tests.

In this review, we highlight the parallels between the performance of ADHD children on tests of working memory, response inhibition, cognitive flexibility, planning, and attention [reviewed by Aguiar et al. (2010)] and the performance of children and laboratory animals on similar tasks after developmental exposure to lead or polychlorinated biphenyls (PCBs). This synthesis builds on several insightful earlier reviews that first drew attention to these commonalities (Cory-Slechta 2003; Rice 1996, 2000).

Human and animal studies of developmental lead or PCB exposure that assessed specific functional domains shown to be impaired in ADHD children were identified via searches of PubMed (http://www.ncbi.nlm.nih.gov/pubmed/) using lead or PCB exposure in combination with the key words “executive function,” “working memory,” “response inhibition,” “attention,” “cognitive function,” “behavior,” and “ADHD,” among others. Because the literature on the effects of lead on cognition in children is so extensive, we included only lead studies published after 1990 that either tested specific neurobehavioral functions relevant to ADHD or directly assessed the relationship between lead exposure and a diagnosis of ADHD. We applied similar criteria to PCB studies, with the exception that we included studies before 1990. For studies of lead in animals, we included those that evaluated effects of developmental exposure on cognitive domains relevant to ADHD and reported steady-state blood lead levels (BLLs) < 100 μg/dL. Findings from animal studies that reported steady state BLLs > 100 μg/dL were not included because they are of questionable relevance to humans. We included PCB studies in animals that evaluated individual noncoplanar congeners or mixtures, but we excluded studies of coplanar PCB congeners because exposure to these congeners results in few cognitive deficits in animal models (Sable and Schantz 2006). Findings were considered statistically significant when defined as such in the original papers, typically with an alpha level of 0.05.

## Lead

### Epidemiologic research

Numerous studies have demonstrated that lead negatively affects children’s cognitive abilities and behavior, and deficits have been observed at BLLs well below the action level of 10 μg/dL set by the Centers for Disease Control and Prevention (CDC 2005). The vast majority of the studies have used measures of general intelligence or intelligence quotient (IQ) as the primary dependent variable (Bellinger 2008; Lanphear et al. 2005). However, some studies have evaluated the association between low-level lead exposure and aspects of executive function and attention that are implicated in ADHD (Table 1). Brief definitions of the tests used in these studies and the behavioral domains the tests assess are provided in Supplemental Material, Table 1 (doi:10.1289/ehp.0901852). Many of these tests are also described in more detail in the context of ADHD in the companion review by Aguiar et al. (2010).

### Lead and executive function

#### Working memory

Meta-analyses report impaired performance on both verbal working memory and nonverbal working memory tests in ADHD children (Aguiar et al. 2010). A handful of epidemiologic studies have assessed the relationship between childhood lead exposure and performance on tests of working memory. These include assessments of prospective birth cohorts in Boston, Massachusetts; Rochester, New York; and Cincinnati, Ohio, as well as several cross-sectional studies. Stiles and Bellinger (1993) assessed working memory in a prospective birth cohort study with detailed information on the children’s lifetime lead exposure. A total of 148 children from an original sample of 249 middle- and upper-class children in Boston were assessed at 10 years of age. The mean BLL of these children was < 8 μg/dL at all ages. Scores on the Wechsler Intelligence Scale for Children–Revised (WISC-R) Freedom from Distractibility Index, which includes arithmetic and digit span and is believed to assess primarily verbal working memory, were inversely associated with BLL at 2 years of age. However, no associations between BLL and immediate recall on either the California Verbal Learning Test for Children (CVLT-C) or the Story Recall test were observed.

Canfield et al. (2003, 2004) examined the association between lifetime average BLL (mean = 7.2; range, 0–20 μg/dL) and performance on tests of spatial working memory in a prospective cohort of 174 children from Rochester, at 4 years of age using the Shape School task and at 5 years of age using the Cambridge Neuropsychological Tests Automated Battery (CANTAB). Few effects were observed at 4 years, but at 5 years children with higher lifetime BLLs showed impaired performance on tests measuring both spatial working memory and spatial memory span. These findings closely parallel the changes seen on the same spatial working memory tasks in ADHD children (Aguiar et al. 2010).

Ris et al. (2004) examined the relationship between childhood BLL and performance on a battery of tests of executive function and attention in 195 children 15–17 years of age from the Cincinnati prospective birth cohort, an inner-city African-American cohort in which 80% of the children had at least one childhood BLL measurement > 15 μg/dL. The investigators used principal components analysis to identify five neuropsychological factors, including a memory factor. However, the memory factor was not related to BLL.

Chiodo et al. (2004) examined the relationship between current BLL and performance on tasks measuring working memory in a cross-sectional study of 237 inner-city African-American children 7.5 years of age from Detroit, Michigan (referred to as Detroit 1 in Table 1). The BLLs of 92% of the children were < 10 μg/dL. BLL was associated with poorer verbal working memory on the Wechsler Intelligence Scale for Children–III (WISC-III) Digit Span and poorer nonverbal working memory on the Seashore Rhythm test, although scores on the WISC-III Freedom from Distractibility Index and the Sternberg Memory test were not affected.

The association between childhood lead exposure and verbal working memory was also evaluated in two cohorts of children from the Netherlands. Minder et al. (1994) did not find a relationship between hair lead and performance on the digit span test in a small cross-sectional study of 43 boys 8–12 years of age enrolled in special education (Amsterdam 1 cohort). In a later study, Minder et al. (1998) tested a larger sample of 313 boys 9–12 years of age enrolled in special education (Amsterdam 2 cohort) and did not find any associations between BLL and performance on either digit span or the Sternberg Memory test. BLLs in these children averaged 4.4 μg/dL (range, 0.8–16.0 μg/dL), which is similar to the BLLs reported in other recent studies. However, these are the only studies to assess the impact of lead exposure in children enrolled in special education. Detecting an additional impact of low-level lead exposure could be more difficult in children with learning disabilities.

A small study of 61 Korean children 7–16 years of age also evaluated the relationship between current BLL and performance on a digit span test (Min et al. 2007). Lead exposure was very low in this cohort, with an average BLL of only 2.9 μg/dL, yet decreased digit span scores indicative of impaired verbal working memory were observed.

Surkan et al. (2007) compared 6- and 10-year-old children whose current BLLs were 5–10 μg/dL with children whose BLLs were 1–2 μg/dL in the New England Children’s Amalgam Trial (NECAT) (*n* = 389). They found that children with higher BLLs had poorer verbal working memory as assessed on the Wide Range Assessment of Memory and Learning (WRAML).

Kordas et al. (2006) assessed the relationship between current BLL and several measures of working memory in 602 first-graders (average BLL, 11.4 μg/dL) from Mexico. They observed a significant negative association only for number correct on the Sternberg Memory test among the ADHD-relevant memory tests they administered. In summary, although some epidemiologic studies of lead exposure have reported an inverse relationship between BLL and performance on working memory tests, unlike ADHD, the findings are somewhat inconsistent across studies and across individual tests within studies.

#### Response inhibition

Impaired response inhibition is another hallmark of ADHD (Aguiar et al. 2010), and the effects of lead on response inhibition have been evaluated in several studies. Walkowiak et al. (1998) evaluated a sample of 384 6-year-old German children on a continuous performance task (CPT) from the Neurobehavioral Evaluation System 2. Current BLL (average, 4.25 μg/dL) was associated with increases in errors of commission (responding to nontarget stimuli), a pattern similar to that exhibited by ADHD children on CPTs (Aguiar et al. 2010). However, BLL was not associated with increased commission errors in either the Detroit 1 cohort (Chiodo et al. 2004) or a second (Detroit 2) cohort of 467 children 7 years of age with current BLLs that averaged 5 μg/dL (Chiodo et al. 2007). In the 2007 study, BLL also was not associated with impulsivity on any of three behavioral rating scales: the Conners Teacher Ratings Scale, Achenbach’s Teacher Report Form, and PROBS-14 Problem Behavior Scale.

Stewart et al. (2006) used an operant schedule frequently used in animal models [differential reinforcement of low rates of responding (DRL)] to evaluate the association between childhood BLL and response inhibition in 167 children from Oswego, New York. They found that BLLs at 2–4 years of age (mean, 4.58 μg/dL) were associated with excessive responding on the DRL task at 9.5 years of age, indicative of impaired response inhibition. These findings resemble those seen in ADHD children tested on a similar schedule, the fixed interval (FI) task (Sagvolden et al. 1998), and in an animal model for ADHD, the spontaneously hypertensive rat, when tested on the FI task (Sagvolden 2000).

#### Cognitive flexibility and planning

The impact of lead exposure on cognitive flexibility has been assessed in a number of studies, most often using the Wisconsin Card Sorting Test (WCST). Increased perseverative responding (persistent, incorrect responding after the response rule has changed) on the WCST was associated with increased BLL in all three studies that used this test (Chiodo et al. 2004; Stiles and Bellinger 1993; Surkan et al. 2007). This parallels the increase in perseverative responding typically observed in ADHD children on the WCST (Aguiar et al. 2010). Poorer performance on the CANTAB Intradimensional–Extradimensional Shift test, a computerized analogue of the WCST, also has been associated with increased BLL (Canfield et al. 2004). However, the results using other tests of cognitive flexibility have not been as consistent. Two lead studies (Amsterdam 1 and NECAT) found no association between BLL and scores on the Stroop Color-Word test (Minder et al. 1994; Surkan et al. 2007), and only one of three studies (Amsterdam 1, but not Amsterdam 2 or NECAT) found an association between BLL and scores on the Trail Making Test Part B (Minder et al. 1994, 1998; Surkan et al. 2007). Interestingly, the performance of ADHD children on the Stroop Color-Word test also is not as clearly or consistently impaired as their performance on the WCST (Aguiar et al. 2010).

Planning is another cognitive domain that is impaired in ADHD children (Aguiar et al. 2010). Although elevated BLL has been associated with deficits in planning in some studies, the results have not been consistent across studies. Increased BLL was associated with increased planning time and a greater number of moves to solve the CANTAB Stockings of Cambridge test, a computerized analogue of the Tower of London (TOL) task, in the Rochester lead cohort (Canfield et al. 2004). However, in the Detroit 1 cohort BLL did not appear to affect performance on the TOL task (Chiodo et al. 2004). This latter finding is in contrast to the impaired performance of ADHD children on the TOL task (Aguiar et al. 2010). Poorer performance on the Rey-Osterrieth Complex Figure Test (ROCF) was associated with higher BLL in the Boston cohort (Stiles and Bellinger 1993), which is consistent with the findings in ADHD children on this task (Aguiar et al. 2010). The ability to solve mazes was not affected by lead exposure in the Amsterdam 1 (Minder et al. 1994) and NECAT (Surkan et al. 2007) cohorts.

### Lead and attention

Vigilance and alertness are two aspects of attention that are impaired in ADHD children, and both appear to be affected by lead exposure. When CPTs were used to assess attention, impaired vigilance, marked by a decrease in the number of correct responses and an increase in omission errors, was associated with BLL in both of the Detroit cohorts (Chiodo et al. 2004, 2007) and in the German cohort (Walkowiak et al. 1998). In contrast, longer reaction times on the CPT, a measure of alertness, were associated with increased BLL in the Detroit 2 but not the Detroit 1 cohort (Chiodo et al. 2004, 2007). Both findings, increased omission errors and increased reaction times, are observed when ADHD children are tested on CPTs (Aguiar et al. 2010). Additionally, for children in the Detroit 2 cohort, BLL was associated with attentional problems on the Conners Teacher Rating Scale (Chiodo et al. 2007). Higher BLL also was associated with higher ADHD and inattention scores on the Barkley–DuPaul ADHD Scale, poorer attention on the Achenbach Checklist, and higher ratings of withdrawn and off-task behaviors on the Barkley Direct Observation Scale for children in the Detroit 2 cohort (Chiodo et al. 2004).

Deficits in alertness are also suggested by associations of BLL or hair lead with longer reaction times while performing several other tests, including simple reaction time tests, the Sternberg Memory task, a mental rotation task, and a stimulus discrimination test in the Detroit 1 (Chiodo et al. 2004), the Amsterdam 1 (Minder et al. 1994), and the Korean (Min et al. 2007) cohorts, but not in the Amsterdam 2 (Minder et al. 1998), NECAT (Surkan et al. 2007), German (Walkowiak et al. 1998), or Mexican (Kordas et al. 2006) cohorts. Finally, Ris et al. (2004) identified a significant association between childhood BLLs and an attention factor identified by principal components analysis, which included CPT omission errors (vigilance) and reaction time (alertness). A stronger association of BLL with attentional problems was observed in boys than in girls. This does not appear to be attributable to differences in exposure because there does not seem to be a sex-related difference in childhood BLL in the United States based on 1988–2004 data from the National Health and Nutrition Examination Survey (NHANES) (Jones et al. 2009). Other epidemiologic studies of lead exposure generally have not reported whether there are sex differences in outcomes, but it is well established that boys are at greater risk for ADHD (Aguiar et al. 2010).

In summary, studies of lead exposure in children provide evidence for impairments in several aspects of executive function and attention that are impaired in ADHD children. The functions most consistently affected across studies include cognitive flexibility, vigilance, and alertness. There is also some evidence that impairments in working memory, planning, and response inhibition are associated with lead exposure, but the findings for these cognitive domains are less consistent across studies.

### Lead and ADHD

In addition to the parallels between the behavioral domains affected in lead-exposed and ADHD children, an association between BLL and a diagnosis of ADHD has been reported in a number of recent studies (Table 2; see also Braun et al. 2006; Froehlich et al. 2009; Ha et al. 2009; Nigg et al. 2008, 2010; Roy et al. 2009; Wang et al. 2008). Importantly, this relationship was reported even at BLLs well below the CDC’s 10-μg/dL action level. No published studies were identified that looked for but did not observe an association between BLL and ADHD, although one study (Ha et al. 2009) reported an association that did not reach the traditional *p* = 0.05 threshold for statistical significance.

A study using the NHANES data (Braun et al. 2006) reported that the prevalence of parent-reported ADHD diagnosis or treatment was four times higher among children with BLL > 2.0 μg/dL compared with those with BLL < 0.8 μg/dL. Additional studies in Korea (Ha et al. 2009), China (Wang et al. 2008), India (Roy et al. 2009), and the United States (Froehlich et al. 2009) also suggest a link between BLL and a diagnosis of ADHD (Table 2).

Although evidence is beginning to emerge linking lead exposure to ADHD even at relatively low levels of exposure, the data are currently insufficient to infer causality. Furthermore, it is not clear whether behaviors exhibited by ADHD children could result in increased exposure to lead rather than (or in addition to) a direct contribution of lead exposure to the pathogenesis of ADHD. At this juncture, we are not aware of any studies that have attempted to address this difficult but important question.

### Animal models

Numerous studies have used rodent and primate models to assess the effects of early lead exposure on tests of executive functions similar to those affected in ADHD children [Table 3; see also reviews by Cory-Slechta (2003) and Rice (1993, 1996, 2000)]. The findings reveal that early lead exposure impairs performance on tasks that assess spatial working memory, response inhibition, cognitive flexibility, and temporal information processing. Attentional processes also seem to be affected, although relatively few animal studies have specifically assessed attention. Adverse effects have been reported at BLLs as low as 11–13 μg/dL (Rice 1993). As summarized above, this is similar to the BLLs associated with adverse effects in children. Descriptions of the various tests used in animal models are given in Supplemental Material, Table 2 (doi:10.1289/ehp.0901852).

### Executive function

#### Working memory

Lead has been shown to impair performance on the delayed spatial alternation (DSA) task, a commonly used test of spatial working memory. Despite differences in experimental procedures, developmentally lead-exposed monkeys in several studies conducted in two different labs were impaired in their ability to learn the DSA task (Levin and Bowman 1986; Levin et al. 1987; Rice and Gilbert 1990a; Rice and Karpinski 1988). In both labs, lead-exposed monkeys were more likely to press the same lever repeatedly (perseverate) rather than alternate between levers. Similar findings were reported in rats exposed to lead as juveniles and tested on a DSA task (Alber and Strupp 1996), although the findings in rats are more inconsistent (Cory-Slechta et al. 1991).

#### Response inhibition

Although the findings are inconsistent in children, impairments of response inhibition after developmental lead exposure are well documented in animal models (Table 3). Early lead exposure increases rates of responding during both FI training and extinction in monkeys [reviewed by Rice (1993)] and during FI training in rats [reviewed by Cory-Slechta (2003)], similar to the findings seen in ADHD children (Sagvolden et al. 1998) and in an animal model of ADHD, the spontaneously hypertensive rat (Sagvolden 2000), when tested on FI tasks. The effects of lead exposure on response inhibition also were examined in lead-exposed monkeys using a DRL schedule in which early responses reset the clock, postponing reinforcement (Rice 1992; Rice and Gilbert 1985). Monkeys with moderate BLLs (11–13 μg/dL) learned the DRL task more slowly, but eventually achieved reinforcement rates indistinguishable from controls. However, monkeys with higher BLLs (33 μg/dL) had fewer reinforced responses and shorter average interresponse times.

FI and DRL schedules examine a different aspect of response inhibition than the stopping tasks often used in ADHD children. On FI and DRL schedules, the subject must withhold responding in order to achieve efficient performance, whereas on stopping tasks the subject must stop a response after it has already been initiated (Aguiar et al. 2010). This aspect of response inhibition has not been examined in studies of lead-exposed humans or animals.

Temporal information processing deficits, which are present in ADHD children (Aguiar et al. 2010), also contribute to efficient performance on both DRL and FI schedules but may be hard to disentangle from response inhibition deficits. In another study that more directly evaluated the ability to estimate time, rats exposed to lead as juveniles were evaluated on a minimum response duration task (Cory-Slechta et al. 1981). Lead-exposed rats had shorter response durations and earned fewer reinforcers. These results in monkeys and rats suggest that, as with ADHD, lead may interfere with the ability to use internal cues to accurately predict time.

#### Cognitive flexibility

Discrimination reversal learning, which has parallels with the WCST, has been used to assess cognitive flexibility in lead-exposed animals. As discussed above, relatively low BLLs have been associated with impairments on the WCST in several human studies (Chiodo et al. 2004; Stiles and Bellinger 1993; Surkan et al. 2007). Impairments on the WCST are also observed in ADHD children (Aguiar et al. 2010).

An early study found that juvenile rhesus monkeys with high BLLs showed impairments on spatial, color, and size reversal learning tasks, with the most striking deficits observed on the first reversal after original learning (Bushnell and Bowman 1979a, 1979b). Subsequently, reversal learning deficits were found in monkeys exposed to lower levels of lead (BLLs of 11–20 μg/dL) [reviewed by Rice (1993)]. Higher BLLs impaired both initial discrimination learning and reversals, whereas the effects of lower BLLs were evident on reversals but not on original learning. Response latencies did not differ between exposed and control monkeys, indicating the effects were not due to increased reaction time (Gilbert and Rice 1987). Reversal learning deficits were also reported in rats exposed to lead chronically from conception (Garavan et al. 2000; Hilson and Strupp 1997), although response pattern analyses suggested that lead-exposed rats exhibited an associative or learning deficit rather than a problem with cognitive flexibility.

Concurrent random interval–random interval (RI-RI) schedules, which allow pressing on two response levers to be reinforced at different frequencies, are another way to evaluate the ability to change response strategies. Lead-exposed monkeys were found to be impaired in their ability to transition between levers in an RI-RI task (Newland et al. 1994).

### Attention

Relatively few animal studies have assessed attentional processes after lead exposure. Performance on vigilance tasks was impaired in rats postnatally exposed to lead (Brockel and Cory-Slechta 1999; Morgan et al. 2001; Stangle et al. 2007). Significant increases in omission errors, indicative of a deficit in vigilance, and premature responses, suggesting impaired inhibitory control, were observed. However, the same rats did not exhibit a selective attention deficit when a distracting stimulus was introduced (Stangle et al. 2007).

### Neurochemistry

Lead-exposed animals also have underlying neurochemical deficits that share commonalities with ADHD, including reduced dopamine signaling (e.g., Cory-Slechta 1997; Levin et al. 1987). However, not all studies are concordant. Zuch et al. (1998) demonstrated that lead may increase dopaminergic signaling in mesolimbic pathways. Changes in dopamine signaling have been shown to play a role in the lead-induced deficits on both FI and DSA tasks, but not in deficits on a repeated acquisition task (Cory-Slechta 1997; Levin et al. 1987). For example, the prefrontal cortex—a brain region that has been implicated in ADHD (Aguiar et al. 2010)—is critical for accurate performance on the DSA task (e.g., Izaki et al. 2008; Sloan et al. 2006), with reductions in prefrontal dopamine producing impairments on short-delay DSA tasks in both rodents and nonhuman primates (Brozoski et al. 1979; Bubser and Schmidt 1990). In line with this, DSA deficits in lead-exposed animals were most pronounced at the shorter delays or were constant across delays (Alber and Strupp 1996; Levin and Bowman 1986). Also, treatment with the dopamine precursor l-dopa ameliorated the lead-induced deficit (Levin et al. 1987).

In summary, the cognitive domains impaired in lead-exposed animals parallel those affected in children exposed to lead (Canfield et al. 2004; Chiodo et al. 2004) and who have ADHD (Aguiar et al. 2010). There are also parallels between the neurochemical alterations underlying the lead-induced cognitive impairments and the neurochemical alterations believed to underlie ADHD (Brennan and Arnsten 2008).

## Polychlorinated Biphenyls

### Epidemiologic research

Researchers of both PCBs (Rice 2000) and ADHD (Nigg 2006) have pointed out that prenatal exposure to PCBs results in behavioral impairments that share significant commonalities with ADHD. In a recent review, Boucher et al. (2009) provide a synthesis of findings indicating that prenatal PCB exposure is associated with deficits on tasks that assess functions deficient in ADHD children. Table 4 presents the findings from epidemiologic studies on PCBs. It is difficult to compare PCB exposure across studies because of the different analytical techniques that have been used and the different subsets of PCB congeners that have been measured. Longnecker et al. (2003) addressed this by comparing PCB exposure across several studies using median concentrations of the most prevalent PCB congener (PCB-153). They found that the median PCB-153 concentrations in studies conducted in Michigan and the Netherlands ranged from 100 to 120 ng/g, whereas the median in the Oswego cohort was lower (40 ng/g). These studies are presented below.

### PCBs and executive function

#### Working memory

Scores on working memory tests appear to be inversely associated with prenatal PCB exposure. In a prospective study, Vreugdenhil et al. (2002) evaluated the performance of 372 Dutch children 6.5 years of age (Netherlands cohort) on the McCarthy Scales of Children’s Abilities (MSCA) Memory Scale. Poorer scores were associated with PCB exposure, but only in formula-fed children of younger mothers and parents with lower verbal IQ scores (*n* = 178), suggesting that children from more disadvantaged backgrounds may be more at risk. In a separate prospective birth cohort from Oswego, Stewart et al. (2008) assessed verbal working memory in 156 of the original 293 children at 9 years of age using the WISC-III. Decreased freedom from distractibility scores, indicative of poorer verbal working memory, were associated with PCB exposure.

Similarly, in a prospective birth cohort from Michigan, deficits on working memory tasks were associated with prenatal PCB exposure at 4 years of age (*n* = 205–219) (Jacobson et al. 1990, 1992) and 11 years of age (*n* = 145–152) (Jacobson and Jacobson 2003). These associations included poorer memory scores on the MSCA at age 4, deficits in number correct on the Sternberg Memory test at both ages and on the WISC-R Digit Span at age 11, and poorer performance on the Seashore Rhythm test, an assessment of nonverbal auditory working memory, at age 11. There was no association between prenatal PCB exposure and scores on the Corsi Spatial Span task, a measure of working memory in the visual-spatial domain. With most tests an association was observed only when the analysis was limited to children who were not breast-fed (*n* = 44–52) (Jacobson and Jacobson 2003). Later the authors published evidence that mothers with higher IQs are both more likely to breast-feed and more likely to provide a better intellectual environment for their children, resulting in fewer adverse outcomes (Jacobson and Jacobson 2006). These findings and those from the Netherlands cohort suggest that other social or environmental factors can modify the effects of environmental exposures.

In conclusion, PCB exposure appears to affect performance on verbal working memory tests, but there has not been sufficient research to determine whether performance on nonverbal working memory tests is affected. By comparison, lead appears to affect performance on working memory tests to a lesser extent, whereas ADHD children are impaired on both verbal and nonverbal working memory tasks, with moderate effect sizes (Aguiar et al. 2010).

#### Response inhibition

Two groups have reported associations between prenatal PCB exposure and deficits in response inhibition. In the Oswego cohort, children with higher cord serum PCB levels made more errors of commission on CPTs at ages 4.5 years (*n* = 197) (Stewart et al. 2003), 8 years (*n* = 182) (Stewart et al. 2005), and 9.5 years (*n* = 183) (Stewart et al. 2005). The clearest evidence of a deficit was observed when the percentage of target stimuli was large relative to nontarget stimuli, indicating that when the children were required to respond more frequently to targets, they were less able to inhibit responding for nontargets. There was also an inverse association at 9.5 years of age between prenatal PCB exposure and interresponse times on a DRL task (Stewart et al. 2006). Children with higher PCB levels tended to respond too soon and earn fewer rewards. These significant findings from the DRL study are concurrent with, but separate from, the findings related to lead exposure in this cohort, as discussed above.

In the Michigan cohort, Jacobson et al. (1992) did not observe an increase in errors of commission on a similar CPT at 4 years of age but did see an increase in commission errors in a version of the Sternberg Memory task at 4 years and on a CPT at 11 years (Jacobson and Jacobson 2003). However, this relationship was only seen in the subset of children who were not breast-fed. ADHD children also make more commission errors on CPTs (Aguiar et al. 2010), and the DRL findings are similar to those of ADHD children performing on an FI schedule (Sagvolden et al. 1998). In contrast, lead exposure has not been associated with an increased frequency of commission errors in children performing CPTs with the exception of the German study (Walkowiak et al. 1998).

#### Cognitive flexibility and planning

Cognitive flexibility and planning have not been well studied in PCB-exposed children. The WCST and the Stroop Color-Word test, both of which assess cognitive flexibility, have been used only in the Michigan study (Jacobson and Jacobson 2003). Children with greater prenatal PCB exposure had more difficulty changing response strategy when the stimulus dimension changed on the WCST, but the finding was significant only in the subset of children that were formula-fed. No significant findings were seen on the Stroop test. These results are similar to those observed in lead-exposed children. ADHD children are impaired on both the WCST and Stroop tests, although the findings on the Stroop test are less consistent (Aguiar et al. 2010). Vreugdenhil et al. (2004a) evaluated 9-year-olds from a subgroup of the Netherlands cohort (*n* = 207) who had the lowest (*n* = 42) and highest (*n* = 41) prenatal PCB exposure on tests of planning ability. Deficits were observed on the TOL task but not on the ROCF in the children with higher exposure. Performance on these and other planning tasks is impaired in ADHD children (Aguiar et al. 2010) and, in some studies, in lead-exposed children (Table 1).

### PCBs and attention

Interestingly, no significant associations have been observed between prenatal PCB exposure and omission errors (a measure of vigilance) on CPTs. This was true for children at different ages: 4.5, 8, and 9.5 years in the Oswego cohort (Stewart et al. 2003, 2005) and 4 and 11 years in the Michigan cohort (Jacobson and Jacobson 2003). This differs from the findings of increased omission errors on CPTs in ADHD children (Aguiar et al. 2010) and in some lead studies (Chiodo et al. 2004, 2007; Walkowiak et al. 1998). In contrast, prenatal exposure to PCBs has been associated with slower reaction times (a measure of alertness) in both the Michigan cohort (Jacobson and Jacobson 2003; Jacobson et al. 1992) and the Netherlands cohort (Vreugdenhil et al. 2004a, 2004b).

In the Michigan cohort, higher prenatal PCB exposure was associated with slower reaction times in a visual discrimination task at 4 years (Jacobson et al. 1992) and in a mental rotation task at 11 years (Jacobson and Jacobson 2003). Additionally, PCB exposure was associated with more errors of omission on a digit cancellation task, which also suggests an impaired ability to stay alert. In contrast, CPT reaction times were not affected at either 4 or 11 years (Jacobson and Jacobson 2003). The differential effects on reaction time across tasks could reflect different information processing requirements of the tasks.

Vreugdenhil et al. (2004a) compared reaction times on a modified version of Letz’s (1994) Simple Reaction Time test in the two groups of 9-year-olds from the Netherlands cohort who had the lowest and highest prenatal PCB exposure. Low-exposure children were significantly faster than high-exposure children. Vreugdenhil et al. (2004b) also measured the magnitude of the P300 event-related potential, which is a neurophysiologic measure of attentional processing time, in the same sample of 9-year-old children during an auditory task. High-exposure children had significantly longer P300 latencies, again suggesting an association between prenatal PCB exposure and slower processing speed. Although these findings are potentially important, they should be interpreted with caution given that findings observed in a subgroup of the original population (i.e., Jacobson and Jacobson 2003; Vreugdenhil et al. 2004a, 2004b) can be difficult to replicate in epidemiologic research.

In summary, PCB-exposed children show evidence of impaired verbal working memory, response inhibition, cognitive flexibility, and alertness on various tasks, but vigilance, as assessed on CPTs, appears to be relatively intact. In comparison, lead-exposed children demonstrate impaired cognitive flexibility, vigilance, and alertness, as well as less consistent deficits in working memory and planning, but only limited effects on response inhibition. Deficits in all of these functions are typical in ADHD children (Aguiar et al. 2010).

### Animal models

A variety of studies have used animal models to assess the effects of PCB exposure on executive functions impaired in ADHD children (Table 5). Many of the same tests used to assess the effects of lead exposure on executive function have been employed, allowing for comparisons across the two contaminants.

### Executive function

#### Working memory

Performance on the DSA working memory task is impaired by early PCB exposure in a number of studies [Levin et al. 1988; Rice and Hayward 1997; Schantz et al. 1995; Widholm et al. 2004; reviewed by Sable and Schantz (2006)], although some studies did not report any effects (Levin et al. 1988; Schantz et al. 1997; Zahalka et al. 2001). Rice and Hayward (1997) found that monkeys treated with an experimental mixture of PCBs in the early postnatal period showed impaired acquisition of the task, responding repeatedly at the same location (perseverating) rather than alternating responses between the two locations. The monkeys also made more errors at short delays. Similar findings were reported in monkeys exposed to the commercial PCB mixture Aroclor 1248 (Levin et al. 1988), in female but not in male rats exposed to individual PCB congeners (Schantz et al. 1995), and in rats of both sexes exposed to Aroclor 1254 (Widholm et al. 2004). The findings of perseverative lever pressing on the DSA task parallel those observed with lead exposure in monkeys and rodents.

#### Response inhibition

Performance on tests of response inhibition including FI and DRL operant schedules is also impaired by early PCB exposure. Rice (1997, 1998) found that the same treated monkeys that were impaired on the DSA task made more responses with shorter interresponse times on both FI and DRL schedules. As described above, excessive responding with shorter interresponse times on a DRL schedule was correlated with both PCB and lead exposure in children from the Oswego cohort (Stewart et al. 2006).

The findings of impaired response inhibition in monkeys have been mirrored in several rodent studies. Lactational exposure of rats to PCB-153 produced increased responding during FI training and extinction in male rats (Holene et al. 1998) but not in female rats (Holene et al. 1999). In a separate study, juvenile male rats exposed to either PCB-contaminated fish or Aroclor 1248 concentrations equivalent to those in the fish (Berger et al. 2001) showed increased lever pressing and bursts of responding on an FI task. Similar findings have been observed in ADHD children tested on FI schedules (Sagvolden et al. 1998). Perinatal exposure of rats to an environmental PCB mixture (Kostyniak et al. 2005) resulted in a decrease in the ratio of reinforced:nonreinforced lever presses on a DRL schedule (Sable et al. 2009), as well as higher rates of responding during an extinction schedule after DRL testing (Sable et al. 2006). The findings of impaired performance on FI and DRL schedules in PCB-exposed animals parallels the findings seen with lead-exposed animals tested on similar schedules.

#### Cognitive flexibility

Discrimination reversal learning tasks have been used to assess the effects of PCBs on cognitive flexibility in monkeys (Bowman et al. 1978; Rice 1998; Rice and Hayward 1997; Schantz et al. 1989) and in rats (Widholm et al. 2001). When deficits were observed, animals tended to be most impaired on the first reversal after original learning (Schantz et al. 1989; Widholm et al. 2001), with error pattern analyses suggesting this was related to increased perseverative responding (Widholm et al. 2001), similar to the increase in perseverative responding on the WCST observed in children with PCB exposure (Jacobson and Jacobson 2003), lead exposure (Chiodo et al. 2004; Stiles and Bellinger 1993; Surkan et al. 2007), and ADHD (Aguiar et al. 2010). Rice (2000) argued that impaired reversal learning seen with lead and, to a lesser extent, PCB exposure can be explained as an inability to alter established response strategies as opposed to an inability to reallocate mental resources in response to changing situational demands. As discussed above, an RI-RI schedule is another way to evaluate the ability to change response strategies. In contrast to lead-exposed monkeys (Newland et al. 1994), PCB-exposed monkeys were not impaired in their ability to transition between levers on an RI-RI task (Rice and Hayward 1999). The findings from both studies are consistent with Rice’s hypothesis if lead impairs the ability to detect the need for changing response strategies to a greater extent than PCBs.

### Attention

Only one study has scrutinized the effects of PCBs on attentional processes. Bushnell et al. (2002) reported that perinatal exposure of rats to Aroclor 1254 did not affect performance on a signal detection task. Additional studies using the five-choice serial reaction time task to assess attention (Robbins 2002) are ongoing in our laboratory.

### Neurochemistry

Neurochemical studies demonstrate that PCBs cause reductions in dopamine in the prefrontal cortex and striatum (Seegal et al. 1991, 1997). PCBs reduce functioning (Fonnum et al. 2006) and decrease expression (Caudle et al. 2006) of both the dopamine transporter and the vesicular monoamine transporter 2 in dopaminergic neurons *in vitro*, resulting in altered synaptic and cytosolic dopamine clearance. Thus, dysregulation of synaptic catecholamines could mediate some of the cognitive impairments observed after PCB exposure. Of interest, Sable et al. (2009) reported that amphetamine disrupted DRL performance less in PCB-exposed rats, providing a behavioral measure of altered brain dopamine function.

In summary, the findings from studies in animal models reveal deficits on tasks that assess many of the same cognitive domains affected by PCB exposure in humans including tests of working memory, response inhibition, and cognitive flexibility. Performance on these same tasks has been reported to be impaired in laboratory animals and children exposed to lead during early development and in ADHD children (Aguiar et al. 2010). However, despite the obvious parallels between the cognitive and neurochemical alterations produced by PCBs and the cognitive and neurochemical impairments observed in ADHD children, the relationship of PCB exposure to a diagnosis of ADHD remains essentially unexplored. One exception is a recent study that reported a positive association between prenatal PCB exposure and ADHD-like behaviors as assessed on the Conners Rating Scale for Teachers (Sagiv et al. 2010).

## Other Environmental Contaminants

Lead was phased out of gasoline and PCBs were phased out of use in electrical equipment in the 1970s. Since that time, environmental concentrations and human body burdens of both “legacy” contaminants have been slowly decreasing. In contrast, human exposure to other chemicals, including brominated flame retardants (Sjodin et al. 2008), bisphenol A (BPA) (Calafat et al. 2008), phthalates (Hauser and Calafat 2005), certain pesticides (Barr et al. 2005), and polyfluoroalkylated chemicals (PFCs) (Jensen and Leffers 2008), has become ubiquitous. The studies reviewed herein demonstrate a risk to children’s neurobehavioral development from lead and PCB exposure. Whether the risks of altered neurodevelopment from PCB exposure will continue into the future is unknown. However, childhood lead exposure is likely to remain an important public health issue because of the lead contamination in older housing in the United States and the significantly higher contamination that still exists in developing countries (Meyer et al. 2008). Although additional research to more fully delineate the role of lead and PCB exposure in the etiology of ADHD would be valuable, it is equally if not more important to gain a better understanding of potential neurodevelopmental effects of other “emerging” contaminants, of which relatively little is known.

Chemicals such as polybrominated diphenyl ethers (PBDEs) and BPA, which have been shown to disrupt dopamine signaling *in vitro* (Jones and Miller 2008; Mariussen and Fonnum 2003), should be investigated as possible contributing factors in ADHD. Although the effects of PBDE exposures in animal studies are observed at higher levels than those to which humans are exposed, some BPA studies have demonstrated adverse effects at levels comparable to those humans encounter daily (Richter et al. 2007; Vandenberg et al. 2010). Studies of attention or executive function after PBDE exposure are limited, but Driscoll et al. (2009) found that mice postnatally exposed to the commercial PBDE mixture DE-71 exhibited impulsivity and inattention, as evidenced by more premature responses and omission errors, respectively, on the five-choice serial reaction time task. Prenatal PBDE exposure also has been associated with impaired vigilance in a study of 62 children 5–6 years of age (Roze et al. 2009). PBDEs have been shown to reduce vesicular and, to a lesser extent, synaptosomal dopamine uptake *in vitro* (Mariussen and Fonnum 2003), paralleling what has been reported with PCBs (Caudle et al. 2006; Fonnum et al. 2006) and further supporting the idea that PBDE exposure could potentially be a risk factor for ADHD. BPA, a component in polycarbonate plastics, has been shown to reduce dopamine synthesis, release, and turnover, as well as dopamine transporter expression, in rodents [reviewed by Jones and Miller (2008)], but only a few studies have evaluated the effects of developmental exposure on cognitive function and none to date has employed tests relevant to ADHD.

A recently published study reported that prenatal exposure to low-molecular-weight phthalates was associated with poorer parent-rated scores on attention and externalizing problems (including impulsivity and hyperactivity) in 188 children 4–9 years of age using the Behavior Assessment System for Children scales (Engel et al. 2010). Preceding this, Kim et al. (2009) reported that higher urine phthalate metabolites at the time of testing in 261 children 8–11 years of age were associated with higher teacher-rated scores on both the inattention and hyperactivity-impulsivity subscales of the ADHD Rating Scale, whereas CPT testing revealed more omission and commission errors, suggestive of impaired vigilance and response inhibition, but no increases in reaction time, suggesting that alertness was not affected.

A study of an inner-city minority population (*n* = 288) reported an association between prenatal chlorpyrifos exposure and scores in the clinical problems range on both the Attention Problems scale and ADHD scale of the Child Behavior Checklist at 3 years of age (Rauh et al. 2006). Although a study of 356 organophosphate-exposed children of farmworkers did not find a significant association with scores on either of these scales at 2 years of age (Eskenazi et al. 2007), the children were followed up at 3.5 years (*n* = 331) and 5 years (*n* = 323), with several significant associations observed at 5 years of age (Marks et al. 2010). Specifically, prenatal organophosphate exposure (as measured by maternal urinary dialkyl metabolites during pregnancy) was associated with increased scores on the Attention Problems scale and ADHD scale of the Child Behavior Checklist and with an increased ADHD confidence index on the Conners Kiddie CPT task. Finally, a cross-sectional study using NHANES data found that parentally reported ADHD was positively associated with urinary organophosphate metabolite levels (Bouchard et al. 2010). Only a few animal studies have addressed effects of pesticides on behavioral end points relevant to ADHD. Adult exposure of rats to chlorpyrifos impaired vigilance on a visual signal detection task (Bushnell et al. 2001; Samsam et al. 2005), whereas acute juvenile exposure transiently impaired performance on a DSA working memory task (Stanton et al. 1994).

Very recently, a positive association between parentally reported ADHD and serum PFC levels was reported based on an analysis of NHANES data (Hoffman et al. 2010). To our knowledge, this is the first study to examine a potential association between childhood PFC exposure and ADHD. A few animal studies have assessed neurobehavioral outcomes after PFC exposure, but we are not aware of any that have directly assessed cognitive functions relevant to ADHD.

Although in this review we focus on environmental contaminants, a number of other potential environmental factors, including premature birth, low birth weight, and psychosocial adversity, are known or suspected to be risk factors in the development of ADHD on their own and could potentially interact with environmental exposures to modify risk (Banerjee et al. 2007; Swanson et al. 2007). Converging evidence from a number of clinical and population-based studies also links both maternal smoking and maternal alcohol consumption during pregnancy with ADHD (Braun et al. 2006; Pennington et al. 2009), with Froehlich et al. (2009) demonstrating that children with combined lead and prenatal tobacco exposure have a greater risk of ADHD than would be predicted if the individual risks were multiplied. Whether other risk factors interact with chemical exposures to increase ADHD risk is an area that should be further explored.

## Conclusions and Future Directions

With the addition of studies conducted in the last decade, the specific profiles of cognitive deficits associated with exposure to lead and PCBs are coming into sharper focus. The deficits associated with both contaminants bear many similarities to the behavioral problems observed in ADHD children. Table 6 presents a comparison of cognitive domains affected in ADHD, lead, and PCBs. In the case of ADHD, confidence in the findings in Table 6 is based on the number of individual studies, the concordance of the findings, and the effect size of meta-analytic studies reviewed by Aguiar et al. (2010). For lead and PCBs, confidence in the findings is based on the number of individual studies and the concordance of findings across studies.

Both lead and PCBs have effects on tests designed to assess working memory, with deficits observed in humans (Boucher et al. 2009; Canfield et al. 2004; Chiodo et al. 2004; Surkan et al. 2007) and animal models (Alber and Strupp 1996; Rice 1993; Sable and Schantz 2006), although performance on working memory tests has not been consistently associated with lead in human studies (Table 1). Also, it is not clear that the deficits observed in animals on working memory tasks are related to actual impairments in working memory. Typically working memory performance will worsen as the delay interval between trials gets longer. However, the deficits seen with lead and PCBs were present even at the shortest delays and did not get more severe as the delay interval increased, suggesting that some other cognitive or behavioral impairment may underlie the deficit.

Interestingly, ADHD children show more pronounced deficits on tests that assess spatial working memory than on those that assess verbal working memory (Aguiar et al. 2010). Few epidemiologic studies of lead or PCBs have employed spatial working memory tasks. An exception is the study by Canfield et al. (2004), in which increased errors on the spatial span and spatial working memory tasks from the CANTAB battery, suggestive of spatial working memory deficits, were found to be associated with higher BLLs. Use of spatial working memory tests could provide additional information about the extent to which the functional domains affected with lead or PCB exposure parallel those seen in ADHD children.

Although uncertainty remains, studies in children and animal models suggest that lead may impair both response inhibition and attentional processes (Chiodo et al. 2004, 2007; Morgan et al. 2001; Stewart et al. 2006), whereas PCBs appear to impair response inhibition to a greater extent than attention (Bushnell et al. 2002; Jacobson and Jacobson 2003; Stewart et al. 2003, 2005, 2006). This distinction is most evident on CPTs, where lead-exposed children tend to exhibit an increase in omission errors (impaired vigilance) with less consistent increases in commission errors (impaired response inhibition), whereas PCB-exposed children show impairments in commission errors and no increase in omission errors or accuracy (Tables 1 and 4). The evidence from animal models also strongly supports that both lead [reviewed by Rice (1996)] and PCBs [reviewed by Sable and Schantz (2006)] impair response inhibition.

In addition to deficits in response inhibition and vigilance on CPTs, ADHD children are also impaired on the stop signal task (Aguiar et al. 2010). Commission errors on CPTs assess the ability to inhibit the initiation of a response, whereas the stop signal task evaluates the ability to stop a response once it has already been initiated (e.g., Walshaw et al. 2010). To our knowledge, this aspect of response inhibition has not been assessed in lead- or PCB-exposed children or animals. Use of this task could provide additional information about the extent to which the deficits in response inhibition after lead or PCB exposure parallel those seen in ADHD children.

Both contaminants also cause impairments on tasks that involve components of both response inhibition and temporal information processing, such as FI and DRL schedules. These findings are consistent across species, from rats to monkeys [see reviews by Cory-Slechta (2003); Rice (1993, 2000); Sable and Schantz (2006)] to humans (Stewart et al. 2006). Performance on temporal processing tasks is also impaired in ADHD children (Aguiar et al. 2010).

Another interesting pattern that emerges when recent human studies are considered together with older animal research is a relatively consistent association between lead exposure and deficits in cognitive flexibility using such tasks as WCST in humans (Chiodo et al. 2004; Stiles and Bellinger 1993; Surkan et al. 2007) and reversal learning in monkeys [reviewed by Rice (1993)] and rats (Garavan et al. 2000; Hilson and Strupp 1997). Cognitive flexibility is also impaired in ADHD children (Aguiar et al. 2010). The one study that assessed cognitive flexibility after PCB exposure found deficits on the WCST in PCB-exposed children (Jacobson and Jacobson 2003). Deficits on reversal learning tasks are also present in PCB-exposed monkeys and rats [reviewed by Sable and Schantz (2006)]. Although the effects of PCBs are somewhat less consistent than for lead, this could relate to differences in the doses and timing of exposure across studies.

To date, most ADHD research has focused on the combined phenotype or has not differentiated among the three phenotypes (Aguiar et al. 2010). Another avenue for future research would be to investigate whether exposures to lead, PCBs, or other chemicals are differentially associated with specific ADHD phenotypes. Exposure to environmental contaminants may contribute to the heterogeneity in the expression of ADHD. An equally important objective will be to investigate whether there are specific gene polymorphisms, epigenetic changes, or other ADHD risk factors (low birth weight, preterm birth, psychosocial stress, male sex) that interact with PCBs, lead, or other chemicals to increase ADHD risk.

Finally, there is a relatively extensive body of literature reporting the effects of neurodevelopmental exposure to lead and PCBs in animals and humans. It is hoped that the knowledge gained from this review of the literature on these two legacy contaminants will assist researchers attempting to understand the potential contribution of emerging contaminants, including PBDEs, BPA, phthalates, pesticides and PFCs, to ADHD risk.

## Figures and Tables

**Table 1:** Childhood lead exposure and performance on tests of functions impaired in ADHD.

Domain/test	Cohort	Age (years)	Outcome^a^	Reference
Working memory				

Verbal				
WRAML	NECAT	6.0–10.0	↓ visual and verbal scores	Surkan et al. 2007
	Detroit 1	7.5	— story memory	Chiodo et al. 2004
Digit span	NECAT	6.0–10.0	—	Surkan et al. 2007
	Amsterdam 1	8.3–12.0	—	Minder et al. 1994
	Amsterdam 2	9.0–12.0	—	Minder et al. 1998
	Detroit 1	7.5	↓ number correct	Chiodo et al. 2004
	Korea	7.0–16.0	↓ number correct	Min et al. 2007
WISC-R	Boston	10.0	↓ freedom from Distractibility scores	Stiles and Bellinger 1993
	Mexico	7.0	— freedom from Distractibility scores	Kordas et al. 2006
WISC-III	Detroit 1	7.5	— freedom from Distractibility scores	Chiodo et al. 2004
CVLT-C	Boston	10.0	— immediate recall, ↑perseverative responses	Stiles and Bellinger 1993
Memory^b^	Cincinnati	15.0–17.0	— composite memory factor	Ris et al. 2004
Story Recall	Boston	10.0	— immediate recall	Stiles and Bellinger 1993
Sternberg^c^	Detroit 1	7.5	— number correct	Chiodo et al. 2004
	Amsterdam 2	9.0–12.0	— number correct	Minder et al. 1998
	Mexico	7.0	↓ number correct	Kordas et al. 2006
Nonverbal				
CANTAB				
Spatial span	Rochester	5.0	↑nontarget errors	Canfield et al. 2004
Spatial working memory			↑total errors	
Visual memory span	Mexico	7.0	—	Kordas et al. 2006
Seashore^d^	Detroit 1	7.5	↓ number correct	Chiodo et al. 2004
Corsi^e^	Detroit 1	7.5	— number correct	Chiodo et al. 2004
Pattern Memory	Germany	6.0	— number correct	Walkowiak et al. 1998

Response inhibition				

CPT	Detroit 1	7.5	— commission errors	Chiodo et al. 2004
	Detroit 2	7.0	— commission errors	Chiodo et al. 2007
	Germany	6.0	↑commission errors	Walkowiak et al. 1998
Visual search	Mexico	7.0	— commission errors	Kordas et al. 2006
CRT	Amsterdam 1	8.3–12.0	— false responses	Minder et al. 1994
DRL	Oswego	9.0	↓ interresponse times	Stewart et al. 2006

Cognitive flexibility				

WCST	Boston	10.0	↑perseverative responses	Stiles and Bellinger 1993
	NECAT	6.0–10.0	↑perseverative responses ↓ categories completed	Surkan et al. 2007
	Detroit 1	7.5	↑perseverative responses ↓ conceptual level responses	Chiodo et al. 2004
Stroop^f^	Amsterdam 1	8–12	— time to complete	Minder et al. 1994
	NECAT	6.0–10.0	— interference score	Surkan et al. 2007
Trails-B^g^	Amsterdam 1	8.3–12.0	↑time to complete	Minder et al. 1994
	Amsterdam 2	9.0–12.0	— time to complete	Minder et al. 1998
	NECAT	6.0–10.0	— time to complete	Surkan et al. 2007
CANTAB ID-ED Shift	Rochester	5.0	↓ stages completed ↓ completion of ED shift	Canfield et al. 2004

Planning				

WISC-R mazes	Amsterdam 1	8.3–12.0	— correct responses	Minder et al. 1994
WISC-III mazes	NECAT	6.0–10.0	— correct responses	Surkan et al. 2007
CANTAB SOC	Rochester	5.0	↑no. moves to solve ↑planning time	Canfield et al. 2004
TOL	Detroit 1	7.5	— no. trials to solve	Chiodo et al. 2004
ROCF	Boston	10.0	↓ copy organization scores	Stiles and Bellinger 1993

Attention				

Vigilance				
CPT	Detroit 1	7.5	↓ number correct	Chiodo et al. 2004
	Detroit 2	7.0	↑omission errors	Chiodo et al. 2007
	Germany	6.0	↑omission errors	Walkowiak et al. 1998
Attention^h^	Cincinnati	15.0–17.0	↓ attention	Ris et al. 2004
Underlining	Amsterdam 1	8.3–12.0	— number correct	Minder et al. 1994
Alertness				
CPT	Detroit 1	7.5	— reaction time	Chiodo et al. 2004
	Detroit 2	7.0	↑reaction time	Chiodo et al. 2007
Sternberg	Detroit 1	7.5	↑reaction time	Chiodo et al. 2004
Mental rotation	Detroit 1	7.5	↑reaction time	Chiodo et al. 2004
SRTT	Amsterdam 1	8.3–12.0	↑reaction time	Minder et al. 1994
	Amsterdam 2	9.0–12.0	— reaction time	Minder et al. 1998
	NECAT	6.0–10.0	— reaction time	Surkan et al. 2007
	Korea	7.0–16.0	↑reaction time	Min et al. 2007
	Germany	6.0	— reaction time	Walkowiak et al. 1998
Stimulus discrimination	Mexico	7.0	— reaction time	Kordas et al. 2006

Abbreviations: CANTAB, Cambridge Neuropsychological Testing Automated Battery; CPT, continuous performance task; CRT, Choice Reaction Time task; CVLT-C, California Verbal Learning Test for Children; DRL, differential reinforcement of low rates of responding; ID-ED, Intradimensional-Extradimensional Shift test; NECAT, New England Children’s Amalgam Trial; ROCF, Rey-Osterrieth Complex Figure Test; SOC, Stockings of Cambridge; SRTT, Simple Reaction Time test; TOL, Tower of London; WCST, Wisconsin Card Sorting Test; WISC-R, Wechsler Intelligence Scales for Children–Revised; WISC-III, Wechsler Intelligence Scales for Children III; WRAML, Wide-Range Assessment of Memory and Learning.

a ↑indicates significant increase associated with lead exposure; ↓ indicates significant decrease; — indicates no association.

b Memory factor identified by principal component analysis; included short and long delay recall from the CVLT and ROCF.

c Sternberg Memory test.

d Seashore Rhythm test.

e The Corsi test is a visual-spatial analogue of the digit span test.

f Stroop Color-Word test.

g Trail Making Test Part B.

h Attention factor identified by principal component analysis; included CPT omission errors, CPT commission errors and hit reaction time.

**Table 2:** Childhood lead exposure and ADHD.

Diagnostic measure	Cohort (n; age)	OR (95% CI) or Outcome^a^	Reference
Current stimulant medication Parent report of ADHD diagnosed by a doctor	NHANES 1999–2002 (4,707; 4–15 years)	4.3 (1.2–14.0)	Braun et al. 2006
*DSM*-IV-TR	NHANES 2001–2004 (2,588; 8–15 years)	2.3 (1.5–3.8)	Froehlich et al. 2009
Conners ADHD scale	Korea (1,778; school age)	1.98 (0.76–5.13) (BLL > 3.5 vs. < 1.0 μg/dL)	Ha et al. 2009
Independent diagnosis by two clinicians	Case–control (150; 8–17 years)	↑BLL in ADHD, combined type compared with non-ADHD children	Nigg et al. 2008
Independent diagnosis by two clinicians	Case–control (236; 6–17 years)	↑BLL in ADHD, combined type compared with non-ADHD children	Nigg et al. 2010
Conners ADHD scale; CADS; BRIEF	India (756; 3–7 years)	↑scores on the CADS ADHD index	Roy et al. 2009
*DSM*-IV-TR	China (case–control) (630 ADHD, 630 control; 4–12 years)	6.0 (4.10–8.77) (BLL ≥ 10 vs. BLL ≤ 5 μg/dL)	Wang et al. 2008

Abbreviations: BRIEF, Behavior Rating Inventory of Executive Function; CADS, Conners ADHD/*Diagnostic and Statistical Manual of Mental Disorders IV* scales; CI, confidence interval; *DSM*-IV-TR, *Diagnostic and Statistical Manual of Mental Disorders*, 4th ed., Text Revision; NHANES, National Health and Nutrition Examination Survey; OR, odds ratio.

a ↑indicates significant increase associated with lead exposure.

**Table 3:** Animal studies of lead effects on cognitive domains affected in ADHD.

Test	Exposure period	BLL (μg/dL)^a^	Effect^b^	Reference
Working memory				

Monkey studies				
DSA	Infant → adult	174 P, 68 SS	↓% correct, ↑errors	Levin and Bowman 1986, Levin et al. 1987^c^
DSA	Infant → adult	32–65 SS	↓ errors	Levin and Bowman 1988
DSA	Infant → juvenile, infant → adult, or juvenile → adult	19–36 P	↑% incorrect all groups	Rice and Gilbert 1990a
DSA	Infant → adult	15–25 P, 11–13 SS	↑% incorrect	Rice and Karpinski 1988
Rat studies				
DSA	Juvenile M^d^,^e^	26–123 P	No effect	Milar et al. 1981
DSA	Juvenile → adult M	23–49 SS	↑% correct, ↓ errors	Cory-Slechta et al. 1991
DSA	Juvenile F	20–36 SS	↓% correct	Alber and Strupp 1996

Response inhibition				

Monkey studies				
DRL	Infant → adult	115 P, 33 SS	↑responding, ↓ IRT	Rice 1992
DRL	Infant → adult	15–25 P, 11–13 SS	↑responding	Rice and Gilbert 1985
FI, FR-FI^f^	Infant → adult	115 P, 33 SS	Infant: ↓ pause time^g^ Juvenile: ↑responding, ↓ IRT, ↑pause time	Rice 1988
FI-EXT^h^	Infant → adult	50–60 P, 20–30 SS	FI: ↑responding, ↓ IRT EXT: ↑responding	Rice et al. 1979
FI, EXT	Infant → adult	32–65 SS	FI: ↑IOC,^i^ EXT: no effect	Mele et al. 1984
FI	Infant → adult	15–25P, 11–13 SS	↓ IRT	Rice 1985b
DSA	Infant → juvenile, infant → adult, or juvenile → adult	19–36 P	↑premature responses, ↑perseverative errors all groups	Rice and Gilbert 1990a
DSA	Infant → adult	15–25 P, 11–13 SS	↑perseverative errors	Rice and Karpinski 1988
Rat studies				
DRL, EXT	Juvenile M^j^	59–186 P	No effect	Kishi et al. 1983
DRL	Juvenile^j^	33–226 P, 15–56 SS	↑responding^k^	Overmann 1977
FI	Juvenile → adult M	14–54 SS	↑responding	Cory-Slechta et al. 1983
FI	Juvenile → adult M	6–43 SS	Low/moderate doses: ↑responding, ↓ pause time High dose: ↓ responding, ↑pause time	Cory-Slechta and Thompson 1979
FI	Juvenile → adult M^l^	23 SS	↓ responding	Cory-Slechta 1990
FI	Juvenile → adult M	13–23 SS	↑responding	Cory-Slechta and Pokora 1991
FI	Juvenile → adult M	10–20 SS	↑responding, ↓ IRT	Cory-Slechta et al. 1985
FI	(1) preconception → lactation or (2) preconception → adult	Not reported	(2) only: ↓ reinforcers	Zenick et al. 1979
FR-wait	Juvenile → adult M	11–29 SS	↑responding, ↓ waiting	Brockel and Cory-Slechta 1998
DSA	Juvenile → adult M	23–49 SS	↓ premature responses	Cory-Slechta et al. 1991
Repeat acq-perform^m^	Juvenile → adult M	25–74 SS	Acquisition only: ↑premature responses	Cohn et al. 1993
Signal det w/distract^n^	Juvenile → adolescent F	40–140 P, 13–31 SS	↑premature responses at higher Pb doses	Stangle et al. 2007
Signal det w/distract	Juvenile → adult M	16–28 SS	↑commission errors at higher Pb dose	Brockel and Cory-Slechta 1999

Cognitive flexibility				

Monkey studies				
Rev Lrn S/NS	Infant → adult	40–90 SS	↑errors, ↑omissions	Bushnell and Bowman 1979b
Rev Lrn S	Infant → juvenile	32–65 SS	No effect on errors	Bushnell and Bowman 1979a
Rev Lrn S/NS	(1) infant → juvenile, (2) infant → adult, or (3) juvenile → adult	19–36 P	• (1) ↑errors S, NS• (2) ↑errors S• (3) ↑errors S, NS	Rice 1990, Rice and Gilbert 1990b
Rev Lrn S	Infant → adult	50–60 P, 20–30 SS	↑errors	Rice and Willes 1979
Rev Lrn S/NS	Infant → adult	15–25 P, 11–13 SS	↑errors	Rice 1985a, Gilbert and Rice 1987
RI-RI	Gestation	21–70 SS^o^	↓ transition rate	Newland et al. 1994
Rat studies				
Rev Lrn NS	Juvenile^j^	33–226 P, 15–56 SS	↓% correct	Overmann 1977
Rev Lrn S/NS	Lactation or lactation → adult M	36–57 P, 37–43 SS	No effect	Hastings et al. 1984
Rev Lrn S/NS	Preconception → adult F	20–36 SS	↑errors	Hilson and Strupp 1997
Rev Lrn NS	Gestation → lactation or lactation F	131–158 P, 12–18 SS	No effect on errors	Garavan et al. 2000
Repeat Acq-Perform	Juvenile → adult M	25–74 SS	↓% correct	Cohn et al. 1993

Vigilance				

Rat studies				
Signal det^p^	Gestation → lactation or lactation F	131–158 P, 12–18 SS	↑omissions	Morgan et al. 2001
Signal det	Juvenile → adolescent F	40–140 P, 13–31 SS	↑omissions at higher Pb dose	Stangle et al. 2007
Signal det	Juvenile → adult M	16–28 SS	↑omission errors at lower Pb dose	Brockel and Cory-Slechta 1999

Selective attention				

Rat studies				
Signal det w/distract	Juvenile → adolescent F	40–140 P, 13–31 SS	No effect	Stangle et al. 2007

Abbreviations: DRL, differential reinforcement of low rates of responding; DSA, delayed spatial alternation; EXT, extinction; FI, fixed interval; IOC, index of curvature; IRT, interresponse times; Rev Lrn S/NS, reversal learning spatial/nonspatial; RI-RI, random interval–random interval.

a P indicates peak and SS indicates steady state BLL. Values for multiple treatment groups are expressed as a range. Control group values are not included.

b ↑indicates significant increase with lead treatment; ↓ indicates significant decrease.

c DSA testing was repeated on the same monkeys 2 years after original testing; abnormalities persisted.

d Male (M) or female (F) symbol indicates only that sex was tested.

e Pups were gavaged from postnatal days 3–30 instead of exposure through nursing.

f Monkeys were tested on an FI schedule as infants and a combined fixed ratio (FR)-FI schedule as juveniles. Exposure continued to adulthood.

g Postreinforcement pause.

h FI-EXT indicates that FI and EXT schedules were alternated within sessions. FI, EXT indicates that EXT schedule was implemented after FI testing.

i Indicates an accelerated pattern of responding.

j Pups were gavaged from postnatal days 3–21 instead of exposure through nursing.

k Overmann (1977) began DRL testing at 67 days, whereas Kishi et al. (1983) began at ~150 days, by which time BLL would have fallen to a greater extent.

l Differed from other FI studies from the same lab in that rats were dosed for longer periods (8 or 11 months) before FI testing started.

m Multiple repeated acquisition-performance schedule.

n Signal detection task with irrelevant (distracting) cues.

o Range of maternal BLLs; offspring BLLs were not evaluated.

p Signal detection task in which the cue to respond occurred at varied intervals.

**Table 4:** Prenatal PCB exposure and performance on tests of functions impaired in ADHD.

Domain/test	Cohort	Age (years)	Outcome^a^	Reference
Working memory				

Verbal				
MSCA	Michigan	4.0	↓ verbal and numerical memory scores	Jacobson et al. 1990
	Netherlands	6.5	↓ memory scale scores^b^	Vreugdenhil et al. 2002
WISC-III	Oswego	9.0	↓ freedom from Distractibility scores	Stewart et al. 2008
WISC-R	Michigan	11.0	↓ digit span scores (FF)	Jacobson and Jacobson 2003
Sternberg	Michigan	4.0, 11.0	↓ number correct (FF)	Jacobson and Jacobson 2003
AVLT	Netherlands	9.0	— short delay recall	Vreugdenhil et al. 2004a^c^
Nonverbal				
Corsi^d^	Michigan	11.0	— number correct	Jacobson and Jacobson 2003
Seashore^e^	Michigan	11.0	↓ number correct (BF)	Jacobson and Jacobson 2003

Response inhibition				

CPT	Oswego	4.5, 8.0, 9.5	↑commission errors	Stewart et al. 2003, 2005
	Michigan	4.0	— commission errors (FF)	Jacobson et al. 1992
	Michigan	11.0	↑commission errors (FF)	Jacobson and Jacobson 2003
Sternberg^f^	Michigan	4.0	↑commission errors	Jacobson and Jacobson 2003
DRL	Oswego	9.5	↓ interresponse times	Stewart et al. 2006

Cognitive flexibility				

WCST	Michigan	11.0	↑perseverative errors (FF) ↓ categories completed (FF)	Jacobson and Jacobson 2003
Stroop^g^	Michigan	11.0	↓ scores (all^h^)	Jacobson and Jacobson 2003

Planning				

TOL	Netherlands	9.0	↑no. trials to solve	Vreugdenhil et al. 2004a^c^
ROCF	Netherlands	9.0	— copy strategy	Vreugdenhil et al. 2004a^c^

Attention				

Vigilance				
CPT	Michigan	4.0, 11.0	— omission errors	Jacobson and Jacobson 2003
	Oswego	4.5, 8.0, 9.5	— response accuracy	Stewart et al. 2003, 2005
Alertness				
CPT	Michigan	4.0, 11.0	— reaction time	Jacobson and Jacobson 2003
	Oswego	4.5, 8.0, 9.5	Reaction time not reported	Stewart et al. 2003, 2005
KVD	Michigan	4.0	↑reaction time	Jacobson et al. 1992
SRTT	Netherlands	9.0	↑reaction time	Vreugdenhil et al. 2004a^c^
Digit cancel^i^	Michigan	11.0	↑omission errors (FF)	Jacobson and Jacobson 2003
Mental rotation	Michigan	11.0	↑reaction time (all)	Jacobson and Jacobson 2003

Abbreviations: AVLT, Auditory-Visual Learning Test; BF, breast-fed; CPT, continuous performance task; DRL, differential reinforcement of low rates of responding; FF, formula-fed; KVD, Kagan Matching Familiar Figures Visual Discrimination task; MCSA, McCarthy Scales of Children’s Abilities; ROCF, Rey-Osterrieth Complex Figure Test; SRTT, Simple Reaction Time test; TOL, Tower of London; WCST, Wisconsin Card Sorting Test; WISC-R, Wechsler Intelligence Scales for Children–Revised; WISC-III, Wechsler Intelligence Scales for Children III.

a Only studies that assessed children on tests of specific functional domains relevant to ADHD are included (↑indicates significant increase associated with PCB exposure; ↓ indicates significant decrease; — indicates no association).

b Association observed only in children whose mothers were younger and whose parents had lower IQ scores.

c Assessed 42 children with low PCB exposure and 41 with high PCB exposure from a subset of 207 children from the original cohort.

d The Corsi test is a visual-spatial analogue of the digit span test.

e Seashore Rhythm test.

f Sternberg Memory test.

g Stroop Color-Word test.

h Both formula-fed and breast-fed.

i Digit cancellation.

**Table 5:** Animal studies of PCB effects on cognitive domains affected in ADHD.

Test	Exposure period	PCBs^a^	Effect^b^	Reference
Working memory				

Monkey studies				
DSA	Preconception → infant	Aroclor 1016	No effect	Levin et al. 1988
DSA	Preconception^c^	Aroclor 1248	↓% correct, ↑errors	Levin et al. 1988
DSA	Infant M^d^	Milk mixture^e^	↑errors	Rice and Hayward 1997
Rat studies				
DSA	Gestation	PCB-28, -118, or -153	F only: ↓ no. correct, ↓ acquisition	Schantz et al. 1995
DSA	Gestation	PCB-95	No effect	Schantz et al. 1997
DSA	Gestation or gestation → lactation^f^	Aroclor 1016	No effect^g^	Zahalka et al. 2001
DSA	Gestation → lactation	Aroclor 1254	No effect^g^	Zahalka et al. 2001
DSA	Preconception → lactation	Aroclor 1254	↓% correct, ↑errors	Widholm et al. 2004

Response inhibition				

Monkey studies				
DRL	Infant	Milk mixture	↑responding,^h^ ↓ IRT	Rice 1998
FI	Preconception → lactation	Aroclor 1248	No effect	Mele et al. 1986
FI w/reinf omissions^i^	Preconception^j^	Aroclor 1248	↑responding^k^	Mele et al. 1986
FI w/reinf omissions	Preconception → lactation^l^	Aroclor 1248	No effect	Mele et al. 1986
FR-FI	Infant M	Milk mixture	↓ IRT, ↓ pause time^m^	Rice 1997
DSA	Infant M	Milk mixture	↑perseverative errors	Rice and Hayward 1997
Rat studies				
DRL, EXT^n^	Preconception → lactation	Fox River mix^o^	DRL: no effect, EXT: ↑responding, ↓ IRT	Sable et al. 2006
DRL	Preconception → lactation	Fox River mix	↓ reinforced: nonreinforced responses	Sable et al. 2009
VI-DRL	Lactation F	PCB-153	↓ IRT	Holene et al. 1999
FI-EXT^p^	Lactation M	PCB-153	FI: ↑responding, ↑perseverative pressing, EXT: ↑responding	Holene et al. 1998
FI-EXT	Lactation F	PCB-153	No effect	Holene et al. 1999
FI	Preconception → adult M	Clophen A30	↑responding in highest dose group	Lilienthal et al. 1990
FI-EXT	Adolescent → adult M	Aroclor 1248	FI & EXT: ↑responding	Berger et al. 2001
FI w/reinf omissions	Gestation → lactation	Aroclor 1254	F only: ↑responding^k^	Taylor et al. 2002

Cognitive flexibility				

Monkey studies				
Rev Lrn S/NS	Preconception → lactation	Aroclor 1016	S: ↓ acquisition, NS: ↑acquisition^q^	Schantz et al. 1989
Rev Lrn S/NS	Preconception^r^	Aroclor 1248	No effect^s^	Schantz et al. 1989
Rev Lrn S/NS	Preconception^t^	Aroclor 1248	NS: ↑acquisition^q^	Schantz et al. 1989
Rev Lrn S/NS	Preconception → lactation	Aroclor 1248	No effect^s^	Schantz et al. 1989
Rev Lrn S/NS	Preconception → lactation^u^	Aroclor 1248	S, NS: ↑errors	Bowman et al. 1978
Rev Lrn S	Infant M	Milk mixture	No effect	Rice 1998
Rev Lrn NS	Infant M	Milk mixture	↑variability in response latencies	Rice and Hayward 1997
RI-RI	Infant M	Milk mixture	No effect	Rice and Hayward 1999
Rat studies				
Rev Lrn S	Gestation → lactation	Aroclor 1254	↑errors^v^	Widholm et al. 2001

Vigilance				

Rat studies				
Signal det^w^	Gestation → lactation	Aroclor 1254	No effect	Bushnell et al. 2002

Abbreviations: DRL, differential reinforcement of low rates of responding; EXT, extinction; FI, fixed interval; FR-FI, fixed ratio–fixed interval schedule; IRT, interresponse time; Rev Lrn S/NS, reversal learning spatial/nonspatial; RI-RI, random interval–random interval; VI-DRL, variable-interval DRL schedule.

a PCB congeners or mixtures used in each study.

b ↑indicates significant increase with PCB treatment; ↓ indicates significant decrease.

c Two different cohorts of monkeys were tested. Cohort 1 dams’ exposure ended 1 year before conception. The same dams were rebred 32 months after exposure for cohort 2.

d Male (M) or female (F) symbol indicates only that sex was tested.

e PCB mixture representative of congeners in human breast milk.

f One group of pups was exposed through nursing, whereas a second group was exposed through nursing and gavaged daily from postnatal day 3–21.

g DSA testing occurred in five sessions over postnatal days 22–23. Rats were younger and were tested for a shorter period than in other DSA studies.

h Only nonreinforced responses were increased.

i FI with reinforcement omissions; on a percentage of correct trials, reinforcers were omitted (w/reinf).

j Offspring of the same dams in the previous FI experiment (Mele et al. 1986). Dams had not received PCBs for 20 months at the time of the second breeding.

k Increased responding was only seen after trials in which reinforcers were omitted.

l Dams received PCB doses that were 20% of those used in the previous study (Mele et al. 1986).

m Postreinforcement pause.

n Extinction schedule implemented after DRL testing.

o PCB mixture representative of congeners in sport-caught fish.

p FI-EXT indicates FI and EXT schedules were alternated within sessions.

q Facilitated acquisition of a shape problem occurred after shape was used as an irrelevant cue in a prior problem. This may represent failure of the PCB-exposed monkeys to learn the irrelevancy of shape in the prior problem.

r The same first cohort of monkeys tested on the DSA task (Levin et al. 1988).

s Irrelevant cues were not employed in this group of monkeys.

t The same second cohort of monkeys tested on the DSA task (Levin et al. 1988).

u Dams in this experiment received PCB doses that were 2.5 times greater than those used in Schantz et al. (1989).

v Male rats had ↑errors on first reversal due to ↑perseverative responses. Females had ↑errors on fourth and fifth reversals, suggesting impaired ability to make new associations.

w Signal detection task in which the cue to respond occurred at varied intervals.

**Table 6:** Comparison of cognitive domains affected in ADHD and by lead and PCBs in humans and laboratory species: degree of confidence in findings.

		Lead		PCBs	
Domain	ADHD	Human	Animal	Human	Animal
Working memory					
Verbal	+++	++		+++	
Nonverbal (incl. spatial)	++++	++	++	—	++
Response inhibition	+++	+	++++	+++	+++
Cognitive flexibility	++	+++	+++	++	++
Planning	++	++		+	
Attention					
Vigilance (sustained)	+++	+++	++	—	—
Alertness	++	+++		++	

Pluses indicate range of degree of confidence, from low (+) to high (++++). — indicates that literature does not support involvement of the domain.
